# Bis(triethyl­ammonium) tetra­chlorido­cobaltate(II)

**DOI:** 10.1107/S1600536812022441

**Published:** 2012-05-31

**Authors:** Reza Azadbakht, Hassan Hadadzadeh, Hadi Amiri Rudbari

**Affiliations:** a Department of Chemistry, Payame Noor University, Tehran, Iran; b Department of Chemistry, Isfahan University of Technology, Isfahan 84156-83111, Iran; c Dipartimento di Chimica Inorganica, Vill. S. Agata, Salita Sperone 31, Universita di Messina 98166 Messina, Italy

## Abstract

The crystal structure of the title compound, (C_6_H_16_N)_2_[CoCl_4_], is comprised of a tetrahedral [CoCl_4_]^2−^ anion and two independent triethyl­ammonium cations. The latter are featureless while the [CoCl_4_]^2−^ anion exhibits typical Co—Cl bond lengths [2.2428 (15)–2.2847 (16) Å] and a Cl—Co—Cl angular range of 107.58 (6)–112.73 (7)°. In the crystal, N—H⋯Cl hydrogen bonds between the two crystallographically independent cations and the [CoCl_4_]^2−^ anion generate discrete ion triplets. The two Co—Cl bonds involved in these inter­actions are slightly longer than the remaining two.

## Related literature

For the crystal structure of a related complex, see: Clegg & Martin (2007 ▶).
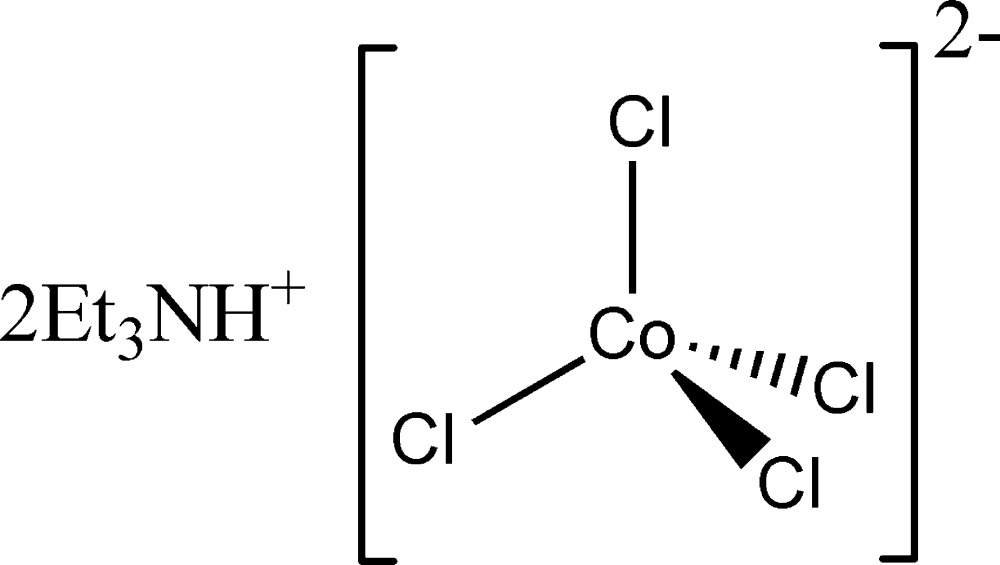


## Experimental

### 

#### Crystal data

(C_6_H_16_N)_2_[CoCl_4_]
*M* *_r_* = 405.13
Orthorhombic, 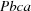

*a* = 11.981 (5) Å
*b* = 13.226 (5) Å
*c* = 25.946 (10) Å
*V* = 4112 (3) Å^3^
*Z* = 8
Mo *K*α radiation
μ = 1.35 mm^−1^
*T* = 296 K
0.18 × 0.14 × 0.13 mm

#### Data collection

Bruker APEXII CCD diffractometer
Absorption correction: multi-scan (*SADABS*; Bruker, 2007 ▶) *T*_min_ = 0.661, *T*_max_ = 0.745
42005 measured reflections
4090 independent reflections
2078 reflections with *I* > 2σ(*I*)
*R*_int_ = 0.117

#### Refinement

*R*[*F* ^2^ > 2σ(*F* ^2^)] = 0.052
*wR*(*F* ^2^) = 0.119
*S* = 1.00
4090 reflections
180 parameters
1 restraint
H atoms treated by a mixture of independent and constrained refinement
Δρ_max_ = 0.45 e Å^−3^
Δρ_min_ = −0.51 e Å^−3^

### 

Data collection: *APEX2* (Bruker, 2007 ▶); cell refinement: *SAINT* (Bruker, 2007 ▶); data reduction: *SAINT*; program(s) used to solve structure: *SHELXS97* (Sheldrick, 2008 ▶); program(s) used to refine structure: *SHELXL97* (Sheldrick, 2008 ▶); molecular graphics: *XP* in *SHELXTL* (Sheldrick, 2008 ▶); software used to prepare material for publication: *SHELXTL*.

## Supplementary Material

Crystal structure: contains datablock(s) I, global. DOI: 10.1107/S1600536812022441/bg2461sup1.cif

Structure factors: contains datablock(s) I. DOI: 10.1107/S1600536812022441/bg2461Isup2.hkl

Additional supplementary materials:  crystallographic information; 3D view; checkCIF report

## Figures and Tables

**Table 1:** Hydrogen-bond geometry (Å, °)

*D*—H⋯*A*	*D*—H	H⋯*A*	*D*⋯*A*	*D*—H⋯*A*
N1—H1⋯Cl4	0.84 (4)	2.39 (5)	3.216 (6)	170 (4)
N2—H2⋯Cl1	0.90 (4)	2.34 (4)	3.214 (5)	163 (4)

**Fig. 1.**
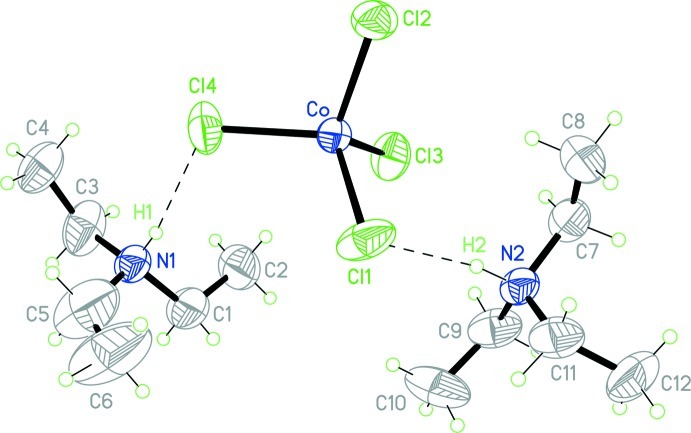
The asymmetric unit with atom labels and 50% probability ellipsoids for non-H atoms. Hydrogen bonds are shown as dashed lines.

**Fig. 2.**
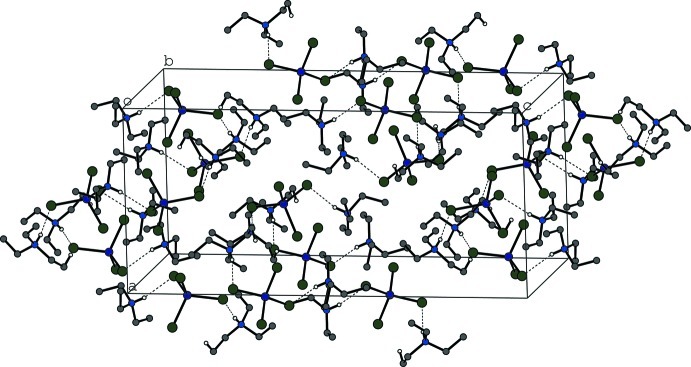
The packing, viewed along the c axis. Hydrogen bonds are shown as dashed lines, and H atoms not involved in hydrogen bonding have been omitted.

## supplementary crystallographic information

### Comment

The crystal structure of the title compound, Bis (triethylammonium) tetrachloridocobalt (II), is comprised of a [CoCl_4_]^2-^ anion and two independent triethylammonium cations. The crystal structure of triethylammonium is similar to that of its analogue salts, Clegg and Martin (2007). The [CoCl_4_]^2-^ anion possesses typical Co—Cl bonds (range: 2.2428 (15) to 2.2847 (16) Å), while the Cl—Co—Cl angles range from 107.58 (6) to 112.73 (7)°. An *ORTEP* diagram of the asymmetric unit is presented in Fig. 1. In the crystal structure intermolecular N—H—Cl hydrogen bonds are observed between each of the two crystallographically independent cations and the tetrahedral [CoCl_4_]^2-^ anion that generate discrete ion triplets (Table 1). The Co—Cl bonds involving the hydrogen-bonded Cl atoms are slightly longer than the remaining two. The hydrogen bonds between the organic cations and the [CoCl_4_]^2-^ anions contribute to the stability of crystal packing (Fig. 2).

### Experimental

The compound was obtained unexpectedly in an unsuccessful attempt to prepare a cobalt(II) macrocyclic Schiff-base complex. To 2,6-diformyl-4-chlorophenol (1 mmol) and CoCl_2_.6H_2_O (2 mmol) in 25ml of MeOH, a methanolic solution (25 ml) of 1,2-bis (2'-aminophenoxy)benzene (1 mmol) and NEt_3_ (1 mmol) was added dropwise. The resulting solution was stirred under reflux for 3 h. The precipitate obtained by partial evaporation of the solution was allowed it to stand overnight in refrigerator and was collected by filtration. Suitable crystals for the X-ray crystal structure determination were grown by recrystallization by diffusion of diethylether in the solution of the complex in acetonitrile

### Refinement

N-bound H atoms were located in a difference map and refined isotropically. Other H atoms were positioned geometrically and refined with a riding model (including free rotation about C—C bonds), with *U*_iso_(H) = 1.2 (1.5 for methyl groups) times *U*_eq_(C). Atoms C5 and C6 from one of the ethyl branches of the N1 cationic groups show large displacement factors, much larger than neighbouring atoms and (due to libration effects) giving rise to a short, non realistic C-C sp^3^···sp^3^ distance of 1.250 (9)Å.

### Crystal data

**Table:**

(C_6_H_16_N)_2_[CoCl_4_]	*F*(000) = 1704
*M**_r_* = 405.13	*D*_x_ = 1.309 Mg m^−^^3^
Orthorhombic, *P**b**c**a*	Mo *K*α radiation, λ = 0.71073 Å
Hall symbol: -P 2ac 2ab	Cell parameters from 3697 reflections
*a* = 11.981 (5) Å	θ = 2.3–19.2°
*b* = 13.226 (5) Å	µ = 1.35 mm^−^^1^
*c* = 25.946 (10) Å	*T* = 296 K
*V* = 4112 (3) Å^3^	Irregular, green
*Z* = 8	0.18 × 0.14 × 0.13 mm

### Data collection

**Table:**

Bruker APEXII CCD diffractometer	4090 independent reflections
Radiation source: fine-focus sealed tube	2078 reflections with *I* > 2σ(*I*)
Graphite monochromator	*R*_int_ = 0.117
φ and ω scans	θ_max_ = 26.1°, θ_min_ = 1.6°
Absorption correction: multi-scan (*SADABS*; Bruker, 2007)	*h* = −14→12
*T*_min_ = 0.661, *T*_max_ = 0.745	*k* = −16→16
42005 measured reflections	*l* = −32→32

### Refinement

**Table:**

Refinement on *F*^2^	Primary atom site location: structure-invariant direct methods
Least-squares matrix: full	Secondary atom site location: difference Fourier map
*R*[*F*^2^ > 2σ(*F*^2^)] = 0.052	Hydrogen site location: inferred from neighbouring sites
*wR*(*F*^2^) = 0.119	H atoms treated by a mixture of independent and constrained refinement
*S* = 1.00	*w* = 1/[σ^2^(*F*_o_^2^) + (0.0298*P*)^2^ + 6.2054*P*] where *P* = (*F*_o_^2^ + 2*F*_c_^2^)/3
4090 reflections	(Δ/σ)_max_ < 0.001
180 parameters	Δρ_max_ = 0.45 e Å^−^^3^
1 restraint	Δρ_min_ = −0.51 e Å^−^^3^

### Special details

**Table:**

Geometry. All s.u.'s (except the s.u. in the dihedral angle between two l.s. planes) are estimated using the full covariance matrix. The cell s.u.'s are taken into account individually in the estimation of s.u.'s in distances, angles and torsion angles; correlations between s.u.'s in cell parameters are only used when they are defined by crystal symmetry. An approximate (isotropic) treatment of cell s.u.'s is used for estimating s.u.'s involving l.s. planes.
Refinement. Refinement of *F*^2^ against ALL reflections. The weighted *R*-factor *wR* and goodness of fit *S* are based on *F*^2^, conventional *R*-factors *R* are based on *F*, with *F* set to zero for negative *F*^2^. The threshold expression of *F*^2^ > 2σ(*F*^2^) is used only for calculating *R*-factors(gt) *etc*. and is not relevant to the choice of reflections for refinement. *R*-factors based on *F*^2^ are statistically about twice as large as those based on *F*, and *R*- factors based on ALL data will be even larger.

### Fractional atomic coordinates and isotropic or equivalent isotropic displacement parameters (Å^2^)

**Table:**

	*x*	*y*	*z*	*U*_iso_*/*U*_eq_	
Co	0.05270 (5)	0.21711 (5)	0.12639 (2)	0.0501 (2)	
Cl1	−0.02174 (14)	0.34750 (11)	0.08048 (6)	0.0963 (6)	
Cl2	−0.06848 (12)	0.08799 (10)	0.12358 (5)	0.0758 (4)	
Cl4	0.08198 (12)	0.25981 (12)	0.21063 (5)	0.0840 (5)	
Cl3	0.21929 (11)	0.17851 (12)	0.09060 (5)	0.0786 (4)	
N1	0.2550 (4)	0.4391 (4)	0.23571 (17)	0.0634 (12)	
N2	0.1187 (4)	0.3092 (3)	−0.02326 (17)	0.0593 (12)	
C2	0.3882 (5)	0.3716 (5)	0.1714 (2)	0.106 (2)	
H2A	0.4369	0.3915	0.1438	0.159*	
H2B	0.3332	0.3252	0.1585	0.159*	
H2C	0.4310	0.3393	0.1980	0.159*	
C1	0.3327 (6)	0.4609 (5)	0.1925 (2)	0.098 (2)	
H1A	0.2913	0.4938	0.1651	0.118*	
H1B	0.3891	0.5080	0.2044	0.118*	
C3	0.3132 (5)	0.4070 (6)	0.2830 (2)	0.106 (2)	
H3A	0.3725	0.3609	0.2734	0.127*	
H3B	0.3478	0.4660	0.2985	0.127*	
C4	0.2438 (5)	0.3579 (5)	0.3221 (2)	0.099 (2)	
H4A	0.2892	0.3396	0.3511	0.149*	
H4B	0.2104	0.2982	0.3078	0.149*	
H4C	0.1862	0.4036	0.3331	0.149*	
C6	0.1254 (8)	0.5774 (8)	0.2169 (4)	0.207 (6)	
H6A	0.0886	0.6314	0.2349	0.311*	
H6B	0.0705	0.5342	0.2013	0.311*	
H6C	0.1727	0.6051	0.1906	0.311*	
C5	0.1830 (8)	0.5274 (7)	0.2479 (4)	0.188 (5)	
H5A	0.1314	0.5041	0.2742	0.225*	
H5B	0.2315	0.5760	0.2647	0.225*	
C10	0.2315 (6)	0.4566 (4)	0.0047 (3)	0.121 (3)	
H10A	0.3066	0.4816	0.0066	0.182*	
H10B	0.2003	0.4532	0.0387	0.182*	
H10C	0.1874	0.5013	−0.0161	0.182*	
C9	0.2319 (5)	0.3537 (5)	−0.0188 (2)	0.0868 (18)	
H9A	0.2651	0.3575	−0.0528	0.104*	
H9B	0.2780	0.3093	0.0020	0.104*	
C7	0.1263 (5)	0.2003 (4)	−0.0402 (2)	0.0769 (17)	
H7A	0.1468	0.1980	−0.0764	0.092*	
H7B	0.1847	0.1669	−0.0207	0.092*	
C8	0.0190 (5)	0.1446 (4)	−0.0327 (2)	0.095 (2)	
H8A	0.0279	0.0758	−0.0437	0.143*	
H8B	−0.0386	0.1763	−0.0527	0.143*	
H8C	−0.0011	0.1460	0.0031	0.143*	
C12	0.0734 (7)	0.3786 (5)	−0.1106 (2)	0.137 (3)	
H12A	0.0194	0.4182	−0.1291	0.206*	
H12B	0.0776	0.3122	−0.1254	0.206*	
H12C	0.1451	0.4107	−0.1128	0.206*	
C11	0.0390 (5)	0.3705 (4)	−0.0550 (2)	0.0858 (18)	
H11A	0.0336	0.4379	−0.0405	0.103*	
H11B	−0.0344	0.3398	−0.0532	0.103*	
H2	0.089 (3)	0.310 (3)	0.0084 (17)	0.052 (14)*	
H1	0.212 (4)	0.394 (4)	0.2253 (18)	0.055 (17)*	

### Atomic displacement parameters (Å^2^)

**Table:**

	*U*^11^	*U*^22^	*U*^33^	*U*^12^	*U*^13^	*U*^23^
Co	0.0547 (4)	0.0482 (4)	0.0476 (3)	−0.0002 (3)	0.0041 (3)	−0.0008 (3)
Cl1	0.1149 (13)	0.0708 (10)	0.1032 (12)	0.0411 (9)	0.0525 (10)	0.0382 (9)
Cl2	0.0832 (10)	0.0699 (9)	0.0741 (9)	−0.0231 (8)	−0.0188 (8)	0.0107 (7)
Cl4	0.0758 (9)	0.1138 (12)	0.0623 (8)	−0.0229 (9)	0.0088 (7)	−0.0328 (8)
Cl3	0.0671 (9)	0.0994 (11)	0.0693 (9)	0.0168 (8)	0.0126 (7)	−0.0066 (8)
N1	0.064 (3)	0.067 (3)	0.059 (3)	0.001 (3)	0.015 (3)	0.003 (2)
N2	0.067 (3)	0.057 (3)	0.053 (3)	0.008 (2)	0.020 (2)	0.005 (2)
C2	0.116 (5)	0.098 (5)	0.104 (5)	−0.016 (4)	0.050 (4)	−0.020 (4)
C1	0.116 (5)	0.089 (5)	0.091 (4)	−0.009 (4)	0.053 (4)	0.002 (4)
C3	0.079 (4)	0.166 (7)	0.072 (4)	−0.027 (5)	−0.013 (4)	−0.005 (4)
C4	0.088 (4)	0.143 (6)	0.066 (4)	−0.001 (4)	−0.009 (4)	0.022 (4)
C6	0.188 (10)	0.214 (11)	0.220 (11)	0.125 (9)	0.093 (8)	0.125 (9)
C5	0.222 (11)	0.164 (8)	0.178 (9)	0.113 (8)	0.118 (8)	0.064 (7)
C10	0.103 (5)	0.063 (4)	0.199 (8)	−0.020 (4)	0.031 (5)	−0.003 (5)
C9	0.067 (4)	0.084 (5)	0.109 (5)	0.001 (3)	0.027 (4)	0.026 (4)
C7	0.099 (5)	0.067 (4)	0.065 (3)	0.029 (3)	0.012 (3)	0.001 (3)
C8	0.118 (5)	0.077 (4)	0.091 (4)	−0.010 (4)	−0.013 (4)	−0.013 (4)
C12	0.220 (9)	0.112 (6)	0.080 (5)	0.032 (6)	−0.019 (5)	0.036 (4)
C11	0.088 (4)	0.062 (4)	0.107 (5)	0.023 (3)	0.003 (4)	0.017 (3)

### Geometric parameters (Å, º)

**Table:**

Co—Cl2	2.2428 (15)	C6—C5	1.250 (9)
Co—Cl3	2.2597 (16)	C6—H6A	0.9600
Co—Cl1	2.2778 (16)	C6—H6B	0.9600
Co—Cl4	2.2847 (16)	C6—H6C	0.9600
N1—C3	1.474 (7)	C5—H5A	0.9700
N1—C1	1.486 (6)	C5—H5B	0.9700
N1—C5	1.486 (8)	C10—C9	1.492 (8)
N1—H1	0.84 (4)	C10—H10A	0.9600
N2—C9	1.483 (7)	C10—H10B	0.9600
N2—C11	1.498 (6)	C10—H10C	0.9600
N2—C7	1.508 (6)	C9—H9A	0.9700
N2—H2	0.90 (4)	C9—H9B	0.9700
C2—C1	1.462 (7)	C7—C8	1.494 (7)
C2—H2A	0.9600	C7—H7A	0.9700
C2—H2B	0.9600	C7—H7B	0.9700
C2—H2C	0.9600	C8—H8A	0.9600
C1—H1A	0.9700	C8—H8B	0.9600
C1—H1B	0.9700	C8—H8C	0.9600
C3—C4	1.464 (7)	C12—C11	1.503 (8)
C3—H3A	0.9700	C12—H12A	0.9600
C3—H3B	0.9700	C12—H12B	0.9600
C4—H4A	0.9600	C12—H12C	0.9600
C4—H4B	0.9600	C11—H11A	0.9700
C4—H4C	0.9600	C11—H11B	0.9700
			
Cl2—Co—Cl3	112.73 (7)	C5—C6—H6C	109.5
Cl2—Co—Cl1	107.82 (7)	H6A—C6—H6C	109.5
Cl3—Co—Cl1	107.58 (6)	H6B—C6—H6C	109.5
Cl2—Co—Cl4	108.58 (6)	C6—C5—N1	126.8 (9)
Cl3—Co—Cl4	108.26 (6)	C6—C5—H5A	105.6
Cl1—Co—Cl4	111.92 (7)	N1—C5—H5A	105.6
C3—N1—C1	112.8 (5)	C6—C5—H5B	105.6
C3—N1—C5	108.9 (6)	N1—C5—H5B	105.6
C1—N1—C5	111.8 (5)	H5A—C5—H5B	106.1
C3—N1—H1	111 (3)	C9—C10—H10A	109.5
C1—N1—H1	106 (3)	C9—C10—H10B	109.5
C5—N1—H1	106 (3)	H10A—C10—H10B	109.5
C9—N2—C11	114.3 (4)	C9—C10—H10C	109.5
C9—N2—C7	110.2 (4)	H10A—C10—H10C	109.5
C11—N2—C7	113.2 (4)	H10B—C10—H10C	109.5
C9—N2—H2	107 (3)	N2—C9—C10	113.0 (5)
C11—N2—H2	104 (3)	N2—C9—H9A	109.0
C7—N2—H2	107 (3)	C10—C9—H9A	109.0
C1—C2—H2A	109.5	N2—C9—H9B	109.0
C1—C2—H2B	109.5	C10—C9—H9B	109.0
H2A—C2—H2B	109.5	H9A—C9—H9B	107.8
C1—C2—H2C	109.5	C8—C7—N2	112.4 (4)
H2A—C2—H2C	109.5	C8—C7—H7A	109.1
H2B—C2—H2C	109.5	N2—C7—H7A	109.1
C2—C1—N1	114.3 (5)	C8—C7—H7B	109.1
C2—C1—H1A	108.7	N2—C7—H7B	109.1
N1—C1—H1A	108.7	H7A—C7—H7B	107.9
C2—C1—H1B	108.7	C7—C8—H8A	109.5
N1—C1—H1B	108.7	C7—C8—H8B	109.5
H1A—C1—H1B	107.6	H8A—C8—H8B	109.5
C4—C3—N1	115.8 (5)	C7—C8—H8C	109.5
C4—C3—H3A	108.3	H8A—C8—H8C	109.5
N1—C3—H3A	108.3	H8B—C8—H8C	109.5
C4—C3—H3B	108.3	C11—C12—H12A	109.5
N1—C3—H3B	108.3	C11—C12—H12B	109.5
H3A—C3—H3B	107.4	H12A—C12—H12B	109.5
C3—C4—H4A	109.5	C11—C12—H12C	109.5
C3—C4—H4B	109.5	H12A—C12—H12C	109.5
H4A—C4—H4B	109.5	H12B—C12—H12C	109.5
C3—C4—H4C	109.5	N2—C11—C12	113.1 (5)
H4A—C4—H4C	109.5	N2—C11—H11A	109.0
H4B—C4—H4C	109.5	C12—C11—H11A	109.0
C5—C6—H6A	109.5	N2—C11—H11B	109.0
C5—C6—H6B	109.5	C12—C11—H11B	109.0
H6A—C6—H6B	109.5	H11A—C11—H11B	107.8
			
C3—N1—C1—C2	69.4 (8)	C11—N2—C9—C10	59.1 (7)
C5—N1—C1—C2	−167.5 (7)	C7—N2—C9—C10	−172.1 (5)
C1—N1—C3—C4	−163.6 (6)	C9—N2—C7—C8	165.6 (5)
C5—N1—C3—C4	71.6 (8)	C11—N2—C7—C8	−65.0 (6)
C3—N1—C5—C6	176.5 (11)	C9—N2—C11—C12	64.2 (7)
C1—N1—C5—C6	51.2 (14)	C7—N2—C11—C12	−63.1 (6)

### Hydrogen-bond geometry (Å, º)

**Table:**

*D*—H···*A*	*D*—H	H···*A*	*D*···*A*	*D*—H···*A*
N1—H1···Cl4	0.84 (4)	2.39 (5)	3.216 (6)	170 (4)
N2—H2···Cl1	0.90 (4)	2.34 (4)	3.214 (5)	163 (4)

## References

[bb1] Bruker (2007). *APEX2*, *SAINT* and *SADABS* Bruker AXS Inc., Madison, Wisconsin, USA.

[bb2] Clegg, W. & Martin, N. C. (2007). *Acta Cryst.* E**63**, m1151.

[bb3] Sheldrick, G. M. (2008). *Acta Cryst.* A**64**, 112–122. 10.1107/S0108767307043930 18156677

